# The therapeutic potential of bone marrow mesenchymal stem cells-derived exosomes for retinal and optic nerve diseases

**DOI:** 10.3389/fcell.2026.1733870

**Published:** 2026-03-04

**Authors:** Mingzhe Shi, Shounan Qi, Feifan Qi, Chenguang Wang

**Affiliations:** Department of Ophthalmology, The Second Hospital of Jilin University, Changchun, Jilin, China

**Keywords:** bone marrow mesenchymal stem cell, exosome, Ischemic retinopathy, optic nerve injury, retina

## Abstract

Bone marrow mesenchymal stem cell-derived exosomes (BMSC-exos) represent a promising cell-free therapeutic strategy that offers significant advantages over cell transplantation in the treatment of retinal and optic nerve diseases. By mediating intercellular communication, these nanovesicles deliver bioactive cargo (miRNAs, proteins, lipids) that target key pathological processes such as neuroinflammation, neuronal apoptosis, vascular dysfunction, and oxidative stress. This review aims to systematically summarize current knowledge on and critically evaluate the therapeutic potential of BMSC-exos for major retinal diseases (e.g., diabetic retinopathy, retinal degeneration, and retinal ischaemia) and optic nerve disorders (e.g., glaucoma and optic nerve injury). We review the biogenesis, cargo composition (especially key neuroprotective factors such as miR-21 and miR-146a), and intercellular communication mechanisms of BMSC-exos. Furthermore, we synthesize evidence describing their multifaceted therapeutic effects–including potent neuroprotective, anti-inflammatory, antiapoptotic, and proangiogenic activities–from *in vitro* and *in vivo* studies across relevant disease models. Crucially, we also discuss the substantial barriers impeding translation, including the intrinsic heterogeneity of exosome preparations which complicates standardization, and the notable absence of active interventional clinical trials for retinal indications due to insufficient long-term safety data. Overall, this review highlights the transformative potential of BMSC-exos for improving visual outcomes, while emphasizing that clinical realization is contingent upon overcoming these critical translational hurdles.

## 1 Introduction

Bone marrow mesenchymal stem cells (BMSCs) are multipotent stromal cells that reside in the bone marrow and are capable of differentiating into osteoblasts, chondrocytes, and adipocytes (Adak et al., 2021). First identified by Friedenstein and colleagues in the 1960s as colony-forming unit-fibroblasts (CFU-Fs), BMSCs have been extensively studied to assess their utility in regenerative medicine (Chiang and Chern., 2022). In addition to their differentiation potential, BMSCs exhibit significant immunomodulatory capabilities, such as the ability to suppress T-cell proliferation, modulate cytokine production, and promote regulatory T-cell (Treg) expansion (Chen et al., 2022). However, BMSC transplantation has several limitations, including low targeted differentiation efficiency, immune rejection, and potential tumorigenicity. Emerging evidence suggests that many of the therapeutic effects of BMSCs are mediated primarily by the extracellular vesicles (EVs) they secrete and the functional molecules contained within them (Ma et al., 2020b).

Exosomes are a heterogeneous subclass of EVs (spanning 30–150 nm in diameter) that are released into the extracellular environment via the endosomal pathway. EVs can be broadly categorized into exosomes, microvesicles (100–1,000 nm), and apoptotic bodies (500–2000 nm) (Phinney and Pittenger., 2017). Importantly, owing to limitations in isolation techniques, including differential ultracentrifugation (a commonly used method), the resulting “exosome” preparations often contain a small fraction of coisolated microvesicles; consequently, modern studies frequently refer to this mixture as “small extracellular vesicles” (sEVs) (Mead et al. 2018b). Exosomes encapsulate a diverse group of biomolecules, including proteins, lipids, mRNAs, microRNAs (miRNAs), and long noncoding RNAs (lncRNAs) which are subsequently transferred to recipient cells to modulate their various functions. Exosome biogenesis involves inwards budding of the endosomal membrane to form intraluminal vesicles (ILVs) within multivesicular bodies (MVBs), followed by MVB fusion with the plasma membrane and exosome release. These vesicles play crucial roles in intercellular communication under physiological and pathological conditions, regulating processes such as immune responses, angiogenesis, cell migration, and tissue repair (Liang et al., 2022; Cui et al., 2021). On the other hand, these vesicles are not merely therapeutic carriers but can also act as pathogenic mediators. Exosomes have been implicated in the progression of diseasesEngin (2021), including participating in destruction of pancreatic β -cells in the developments of diabetes complications (Guay et al., 2015, Guay et al., 2012) and contain pathogenic proteins in Alzheimer’s disease (Zheng et al., 2017). Furthermore, exosomes carrying the non-proteolyzed form of Fas ligand (mFasL) can trigger T-lymphocyte apoptosis, potentially inducing immune dysregulation (Alonso et al., 2011). BMSC-exos have been shown to induce many of the therapeutic effects of their parent cells, such as anti-inflammatory, proangiogenic, and tissue repair properties in preclinical models (Ebrahim et al., 2022; You et al., 2025). Importantly, BMSC-exos have demonstrated advantages over whole-cell therapies, including lower immunogenicity, greater stability, simplified storage, and potential for targeted delivery. Furthermore, because they can function within retinal tissue for extended periods and selectively act on retinal cells, exosomes have potential advantages particularly in the treatment of retinal diseases (Mathew et al., 2021). However, the clinical translation of exosome therapies faces several major challenges, including the standardization of exosome isolation and characterization, the incomplete understanding of their precise mechanisms of action, and the critical need for translation from promising preclinical results to clinical validation in humans.

Retinal diseases are disorders that affect the retina, the neural tissue responsible for phototransduction and the transmission of visual signals to the brain via the optic nerve. Key retinal cell types include photoreceptors, bipolar cells, and retinal ganglion cells (RGCs). Refractory conditions such as age-related macular degeneration (AMD), proliferative retinopathies, and ischaemic retinal injuries cause significant visual impairment but currently lack effective treatments (Liu et al., 2020). The immune-privileged status and susceptibility of the retina to inflammation, oxidative stress, and neuronal apoptosis make it a prime target for the multifaceted therapeutic effects of BMSC-exos (Luodan et al., 2024).

Although exosomes derived from alternative MSC sources–including adipose tissue (ADSCs), umbilical cord (UCMSCs), and gingiva (GMSCs)–offer advantages such as ease of access, BMSCs as the earliest found MSCs, their exosomes possess the most extensive evidence base regarding neuroprotection and tissue repair (Li et al., 2023). Consequently, this article reviews recent advances in BMSC-exos based therapy for treating retinal diseases. We summarize the biological characteristics, functional mechanisms, and therapeutic potential of these exosome and provide a comprehensive overview of BMSC-exos as a promising alternative with potential safety advantages to BMSC transplantation (Mead and Tomarev., 2017).

## 2 Cell biological characteristics of exosomes

### 2.1 Structure

Exosomes are a subclass of EVs secreted by many types of cells. First identified in sheep reticulocytes by Pan and Johnstone in 1983, these vesicles were formally termed “exosomes” by Johnstone’s group in 1987. Modern definitions specify that exosomes are cup-shaped EVs that range from 30 to 150 nm in diameter and originate from the endosomal pathway (Liu et al., 2020).

Exosomal formation involves several steps. First, the plasma membrane invaginates, enveloping extracellular components and membrane proteins and forming early sorting endosomes (ESEs). These ESEs can exchange materials with other organelles or fuse with each other to form late-sorting endosomes (LSEs). The endosomal membrane subsequently buds inwards, generating ILVs within MVBs. The MVBs subsequently undergo one of two processes: (1) lysosomal degradation through fusion with autophagosomes or lysosomes (2) exosomal release via plasma membrane fusion and ILV secretion (Hessvik and Llorente., 2018).

The structure of the exosome consists of a lipid bilayer membrane enriched with tetraspanins (CD9, CD63, and CD81), the endosomal sorting complexes required for transport (ESCRTs) (ALIX and TSG101), and cell type-specific surface markers. The cargo of these exosomes can include proteins, lipids, and nucleic acids, predominantly mature microRNAs (miRNAs) along with smaller quantities of long noncoding RNAs (lncRNAs) and mRNAs. Following uptake by recipient cells, exosomal miRNAs regulate gene expression through target mRNA degradation or translational repression, thereby mediating intercellular communication and functional modulation (Mathew et al., 2023).

### 2.2 Intercellular communication

Exosomal release is regulated by several mechanisms. The Rab GTPase family regulates ESCRTs (Hrs, CHMP4, VPS4) and controls MVB transport and fusion with the plasma membrane. Other proteins, such as the Syndecan-syntenin-ALIX complex, nSMase2, and PLD2, are also involved in exosomal release (Hessvik and Llorente., 2018).

Exosomes reach the vicinity of target cells through paracrine activity and allow communication between cells via three primary mechanisms.

Through receptor-mediated signalling pathways, relevant molecules contained within the exosomes are released and bind to receptors on the surface of the target cell membrane, whereby they produce biological effects by activating secondary messengers and triggering signalling cascades.

Additionally, exosomes can bind to specific surface proteins on the target cell membrane, triggering membrane fusion and directly merging with the target cell membrane. Finally, endocytosis allows exosomes to be internalized and subsequently release their contents into the cytoplasm, where they exert their biological effects (Hessvik and Llorente., 2018). In addition, some evidence suggests the involvement of clathrin- and caveolin-dependent endocytosis pathways, potentially mediated by heparan sulfate proteoglycans on the target cell surface (Mathew et al., 2019).

In the retina, exosome endocytosis mechanisms remain incompletely characterized. Uptake efficiency varies across different retinal cells and is temperature-dependent, suggesting the involvement of energy-dependent processes (Mathew et al., 2019). Surface protein interactions between exosomes (e.g., integrins) and target cells (e.g., ICAM-1) play a key role in the internalization rate (Mulcahy et al., 2014). Recent evidence indicates that the actin cytoskeleton is involved during exosome internalization, with exosomes colocalizing with F-actin at sites of membrane invagination (Jiang et al., 2020).

BMSC-exos demonstrate prolonged bioactivity ( >28 days) within RGCs, microglia, and astrocytes following their uptake. Surface engineering strategies, such as chemical modification or antibody conjugation have shown promise in improving cell type-specific delivery, although translational applications require further refinement (Stranford and Leonard., 2017). Moreover, elucidating the precise uptake mechanisms remains essential for developing targeted EV-based therapies for retinal disorders.

## 3 Functional molecules in BMSC-derived exosomes

BMSC-exos contain key functional molecules, including neurotrophic factors (NTFs), miRNAs (which regulate posttranscriptional gene expression), and cytokines (essential for intercellular signalling). These molecular components collectively mediate the therapeutic potential of these exosomes, facilitating tissue regeneration and immunomodulation while producing anti-inflammatory, antiapoptotic, and neuroprotective effects, all of which address key elements in the pathogenesis of retinal disorders (Table 1).

**TABLE 1:** Inclusion and treatment functions of BMSC-exos.

Exosomal cargo	Target pathway/Protein	Functional outcomes	Disease model	Reference
miR-486-3p	TLR4/NF- κ B	Reduced inflammation, enhanced Müller cell survival	DR	Li et al. (2021)
miR-150-5p	MEKK3/JNK/c-jun	Suppressed RGC apoptosis, inhibited microglial activation	IRI	Wang et al. (2023)
miR-21-5p	PDCD4	Attenuated photoreceptor apoptosis	RDD	Deng et al. (2021)
BDNF/NGF	PI3K/Akt	Promoted RGC survival, enhanced axon regeneration	ONC	Cui et al. (2021)
miR-424	Not reported	Decreased RGC apoptosis, suppressed inflammation	IRI	Mathew et al. (2023)
miR-133b-3p	FBN1	Inhibited angiogenesis, ameliorated ROS injury	DR	Liang et al. (2022)
SNHG7	miR-34a-5p/XBP1	Suppressed Müllerller cell apoptosis, mitigated ROS injury, attenuated inflammation, enhanced proliferation	DR	Cao et al. (2021)
miR-129-5p and miR-34a	Wnt/ β -catenin	Alleviated oxidative stress, reduced inflammation, inhibited angiogenesis	DR	Ebrahim et al. (2022)
miR-423-5p	IL-17, TNF, NF- κ B and HIF-1 pathway	Attenuated RGC injury, suppressed inflammation, inhibited microglial activation	ONC	You et al. (2025)

Abbreviations: RGCs, Retinal Ganglion Cells; ONC, Optic Nerve Crush; IRI, Ischemia-Reperfusion Injury; RDD, Retinal Degenerative Disease; DR, Diabetic Retinopathy; ROS, Reactive Oxygen Species.

BMSC-exos contain numerous miRNAs, such as miR-21, miR-30, miR-125b, miR-130a, miR-199a, miR-210, miR-223, and miR-242 (Wang et al., 2017; Quaglia et al., 2022) that modulate target cells through three primary mechanisms: (1) binding to the 3′ untranslated region (3′UTR) of the target mRNA to silence gene expression and block translation; (2) directing mRNA cleavage and degradation to repress protein synthesis; and (3) promoting mRNA deadenylation, leading to decay. By regulating key mRNAs, exosomal miRNAs can critically influence signalling pathways implicated in retinal angiogenesis, inflammation, and neuronal survival (e.g., pathways involving vascular endothelial growth factor (VEGF), nuclear factor kappa-light-chain-enhancer of activated B cells (NF- κ B), and phosphatidylinositol 3-kinase/protein kinase B (PI3K/Akt)), thereby contributing to their therapeutic efficacy in conditions such as diabetic retinopathy (DR) and ischaemia–reperfusion injury (IRI).

Furthermore, BMSC-exos transport a repertoire of NTFs vital for neuronal health and regeneration, including ciliary neurotrophic factor (CNTF), brain-derived neurotrophic factor (BDNF), glial cell line-derived neurotrophic factor (GDNF), platelet-derived growth factor (PDGF), nerve growth factor (NGF), neurotrophin-3 and neurotrophin-4/5 (NT-3, NT-4/5) Mead et al. (2015), insulin-like growth factor 1 (IGF-1), basic fibroblast growth factor (bFGF/FGF2), pigment epithelium-derived factor (PEDF), and erythropoietin (EPO) (Usategui-Martín et al., 2020). These factors specifically support the functionality of RGCs, photoreceptors, and the retinal pigment epithelium (RPE). In particular, the binding of BDNF, a neurotrophin that is highly expressed in glaucomatous retinas, to its receptors (tropomyosin receptor kinase B (TrkB) and the pan-neurotrophin p75NTR) induces activation of c-jun and suppression of caspase-2, which prevents apoptosis and promotes RGC survival (Roubelakis et al., 2007). Elucidating the individual and synergistic actions of the highly heterogenous molecular cargo within BMSC-exos will accelerate the development of targeted regenerative therapies for retinal degenerative diseases (RDDs) and optic neuroinjuries (An et al., 2025).

## 4 Biological functions of BMSC-exos

### 4.1 Anti-inflammatory effects

BMSC-exos exert potent anti-inflammatory effects in ocular disease models, primarily through the suppression of immune cell activation and the regulation of cytokine levels.

Treatment with BMSC-exos has been shown to inhibit the activation and cytokine production of T cells, B cells, and natural killer cells. Furthermore, BMSC-exos can modulate macrophage and microglial polarization (Ni et al., 2019; Morrison et al., 2017), and the results of recent research have suggested that BMSC-exos promote the transition of microglia from the proinflammatory M1 phenotype to the anti-inflammatory M2 phenotype in neuroinflammation models (Jaimes et al., 2017; Spellicy and Stice., 2021). Additionally, You et al. (2025) demonstrated that BMSC-exos inhibit the transition of microglia into the injury-responsive microglia (IRM) phenotype.

By regulating microglia and macrophage activation states, BMSC-exos can attenuate immunopathology through complementary actions on key cellular components of aberrant immune responses, a key mechanism underlying the synaptogenesis and remyelination effects of these exosomes.

The cytokine-modulating effects of BMSC-exos can be categorized into four groups: 1. Suppression of proinflammatory mediators (e.g., TNF- α , COX-2, and IL-1 β ) (Wang et al., 2020; You et al., 2025; Saccu et al., 2022). Mathew et al. (2019), which act through signalling pathways such as the NF- κ B pathway. 2. Downregulation of angiogenic factors (e.g., VEGF-A) (Saccu et al., 2022; Wang et al., 2020; Khiemt et al., 2017), which contributes to inflammation in specific tissues and diseases. 3. Reduction in the expression of adhesion molecules (e.g., ICAM-1 and MCP-1) (Wang et al., 2020; Moisseiev et al., 2017). Yu et al. (2016), which can attract leukocytes and other immune cells to sites of inflammation (Moisseiev et al., 2017) Yu et al. (2016), thereby limiting leukocyte recruitment. 4. Delivery of anti-inflammatory proteins (e.g., IL-10) (Harrell et al., 2018).

The NF- κ B transcription factor complex controls inflammation and immune responses. Li et al. (2021) reported that miR-486-3p in BMSC-exos suppresses TLR4/NF- κ B signalling, suppressing TNF- α , IL-1 β and IL-6 in M uller cells.

Wang et al. (2023) demonstrated that miR-150-5p in BMSC-exos suppresses the MEKK3/JNK/c-jun pathway, reducing IL-6/TNF- α levels and microglial activation in retinal ischaemia models. Thus, BMSC-exos can help orchestrate multifaceted interactions with inflammatory regulators, resulting in coordinated immunosuppression and tissue protection. Identifying additional anti-inflammatory molecules within BMSC-exos and their respective targets could enable selective improvements in vesicle-based immunomodulatory therapies for inflammatory retinal diseases.

### 4.2 Anti-apoptotic effects

BMSC-exos exert potent antiapoptotic effects through multiple molecular mechanisms. Specifically, studies have demonstrated that BMSC-exos suppress the activation of apoptotic caspase-3 and caspase-7 through multiple signalling pathways, thereby inhibiting apoptosis Mathew et al. (2023). This antiapoptotic activity is conferred to multiple retinal cell types, including RGCs, photoreceptors, and M uller cells (Cui et al., 2021; Park et al., 2021).

Mechanistically, BMSC-exos deliver key bioactive molecules that mediate these effects:

1. S1PR1 activates the JAK/STAT3 signalling pathway, counteracting RGC apoptosis (Saccu et al. 2022).
2. Neurotrophins (BDNF/NGF) bind to TrkB receptors, activating the prosurvival PI3K/Akt signalling cascade. This leads to the upregulation of Bcl-2 and the downregulation of Bax and caspase-3, promoting cell viability (Cui et al. 2021; Mead and Tomarev., 2017).
3. miR-486-3p protects M uller cells and RGCs from hyperglycaemia-induced apoptosis via modulation of the TLR4/NF- κ B signalling pathway (Li et al., 2021).
4. miR-21 inhibits photoreceptor apoptosis by directly targeting PDCD4 (Deng et al., 2021).

Thus, BMSC-exos orchestrate concerted antiapoptotic activity across different retinal cell types affected by inflammatory, metabolic, or oxidative stressors (An et al. 2025). This multitargeted mechanism underscores the significant therapeutic potential of BMSC-exos for preserving retinal and neuronal integrity in degenerative pathologies (Liu et al., 2022).

### 4.3 Neuroprotective effects

BMSC-exos exert neuroprotective effects through multiple mechanisms primarily involving anti-inflammatory activity and apoptosis inhibition. Usategui-Martín et al. (2020) demonstrated that human BMSC-exos cocultured with neuroretinal explants induced the upregulation of key neuroprotective factors, including DLL4, nestin, NPTX1, and NPTXR, that contribute to neuroprotection via the promotion of neuronal survival, plasticity, and synaptic function. Furthermore, BMSC-exos deliver neurotrophic factors and miRNAs that bind to cognate receptors on neural cells, thereby increasing neuronal survival, differentiation, and axon growth (Salehi et al., 2017).

Moreover, BMSC-exos have demonstrated potent neuroprotective and pro-regenerative effects in animal models of optic nerve disorders. In experimental glaucoma and multiple sclerosis-related optic neuritis, BMSC-exos treatment prevented neuronal loss and induced axonal remyelination within the optic nerve (Aneesh et al., 2021).

The ability of BMSC-exos to stimulate axon regeneration likely involves mechanisms analogous to those identified in spinal cord injury models, in which BMSC-exos promote axonal regeneration by delivering miRNAs that target phosphatase and tensin homolog (PTEN) (Tang., 2018). PTEN is a critical negative regulator of the mechanistic target of rapamycin (mTOR) pathway, a central signalling cascade essential for neuronal growth and axon regeneration; consequently, BMSC-exos–mediated PTEN inhibition alleviates this intrinsic negative regulation and activates pro-regenerative signalling pathways (Tang, 2018; Sun et al., 2019).

Further supporting the critical roles played by the miRNA cargoes of BMSC-exos, Mead and Tomarev (2017) reported that knockdown of Argonaute-2 (Ago2), a key component of the miRNA-induced silencing complex, significantly impaired the neuroprotective effect of BMSC-exos on RGCs. This finding strongly implicates the role of specific miRNAs within BMSC-exos as mediators of neuroprotection.

Moreover, a variety of studies have demonstrated that BMSC-exos are internalized by RGCs, where they promote neuroprotective functions over 14 days (You et al., 2025; Mathew et al., 2021). In summary, BMSC-exos therapy promotes neuroprotection and axon regeneration within the visual system, partially by counteracting the intrinsic PTEN-mediated suppression of regenerative pathways. Experimental evidence indicates that BMSC-exos may additionally mediate neuroprotective and regenerative effects in the retina and optic nerve by stimulating the production of synergistic neurotrophic, anti-inflammatory, and homeostasis-regulating factors. Further investigations for characterizing the precise pro-regenerative molecular cargo of BMSC-exos and validating their efficacy in diverse optic nerve disease models are essential.

### 4.4 Ischaemia prevention

BMSC-exos confer substantial protection against retinal damage in retinal ischaemic injury models. Previous studies have shown that the intravitreal administration of BMSCs suppresses apoptosis, preserves endogenous repair mechanisms, and mitigates inflammation and vascular permeability in rodent retinal ischaemia models (Johnson et al., 2010), suggesting that paracrine factors mediate these effects. BMSC-exos specifically prevent ischaemic damage and minimize cell death in retinal cells subjected to oxygen–glucose deprivation *in vitro*. Furthermore, intravitreal injection of BMSC-exos improves electroretinogram (ERG) amplitude recovery and diminishes apoptosis across multiple retinal layers following IRI *in vivo* (Mathew et al., 2019). The results of a study by Mathew et al. (2023) indicate that BMSC-exos mitigate the ischaemia-induced dysregulation of apoptosis mediators, including caspase-3/7, Bax, Bcl-2, and p53, potentially through suppression of the JAK/STAT and MAPK signalling pathways. Collectively, these findings demonstrate that BMSC-exos provide cytoprotection and functional preservation in ischaemic retinopathies by influencing inflammatory responses, apoptotic pathways, and prosurvival mechanisms in a coordinated manner.

### 4.5 Angiogenesis regulation

**FIGURE 1:**
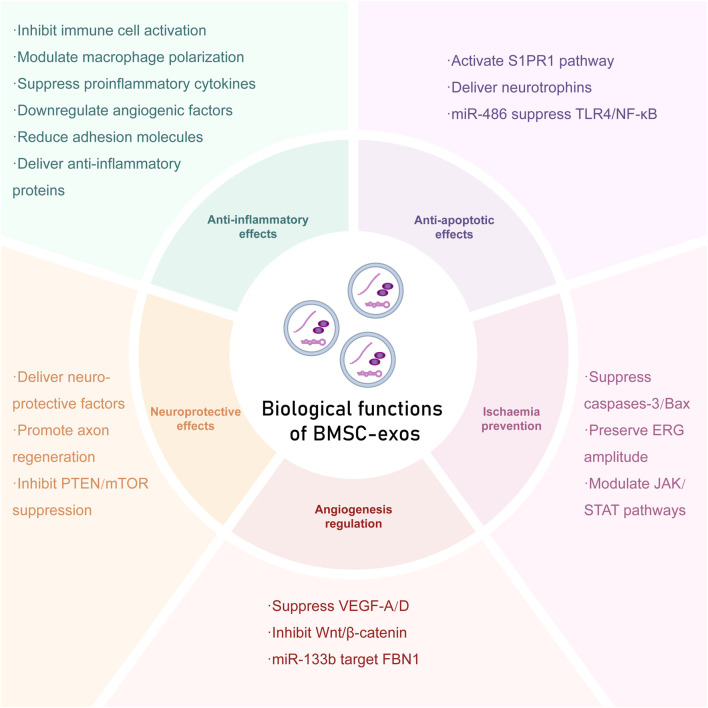
Biological functions of BMSC-exos

BMSC-exos regulate angiogenesis through the induction of pro-regenerative factors. Saccu et al. (2022) demonstrated that BMSC-exos suppress angiogenesis by inhibiting the upregulation of vascular endothelial growth factors (e.g., VEGF-A and VEGF-D). Ebrahim et al. (2022) further demonstrated that BMSC-exos attenuate angiogenesis and vascular leakage by suppressing the Wnt/ β -catenin pathway, which leads to VEGF-A downregulation, and upregulating miR-129-5p and miR-34a in a DR model. Liang et al. (2022) also suggested that BMSC-exos suppress fibrillin-1 (FBN1) to inhibit VEGF/COX-2-driven angiogenesis via the miR-133b-3p/FBN1 axis in diabetic retinopathy models. Notably, compared with exosomes from other MSC sources, BMSC-exos exhibit a distinct miRNA profile, particularly a lack of proangiogenic miRNAs that are regulated by hypoxia-inducible factor-1 (HIF-1), such as miR-210 and miR-378 (Pomatto et al., 2021). This selective cargo composition may prevent the aberrant neovascularization associated with pathologic angiogenesis in conditions such as proliferative diabetic retinopathy. Consequently, BMSC-exos represent a targeted therapeutic approach for regulating physiological angiogenesis and promoting controlled tissue repair in retinal pathologies (Ebrahim et al., 2022) (Figure 1).

## 5 Therapeutic effects on retinal and optic nerve diseases

Exosomes are considered effective treatments for retinal diseases because of their ability to bind type I collagen and persist long term within the vitreous body and retina (Mathew et al., 2019). This section reviews recent research on the application of BMSC-exos in retinal diseases (Table 2).

**TABLE 2:** Summary of BMSC-exos therapies and molecular mechanisms in retinal and optic nerve diseases.

Type of EV	Disease	Target tissue/Cells	Molecular mechanism	Signalling pathway	Effect	Reference
Rat BMSC-exo	ONC	RGCs, microglia	IL-17 ↓ , TNF ↓ , NF- κ B ↓ , HIF-1 ↓	IL-17, TNF, NF- κ B, HIF-1 pathway, ribosome pathway	Protected RGCs from injury, inhibited inflammation, ribosome activity and the transition of microglia into the IRM phenotype	You et al. (2025)
mBMSC-exo	ONC	RGCs	Bcl-2 ↑ , Bax ↓ , caspase-3 ↓ , TNF- α↓ , IL-1 β↓ , IL-8 ↓ , MCP-1 ↓ , AKT phosphorylation ↑	PI3K/AKT	Inhibited RGC apoptosis, downregulated cytokines	Cui et al. (2021)
hBMSC-exo	ONC	NFL and RGCs	Not reported	Not reported	Inhibited RGC apoptosis, promoted neuroprotection	Mead and Tomarev. (2017)
hBMSC-sEV	GLC	RGCs	Not reported	Not reported	Inhibited RGC apoptosis, reduced axonal damage	Mead et al. (2018a)
hBMSC-sEV	GLC	NFL and RGCs	Not reported	Not reported	Inhibited RGC apoptosis, prevented NFL loss	Mead et al. (2018b)
Sca-1+ mBMSC-exo	IRI	RGCs, microglia	MEKK3/JNK ↓ , TNF- α↓ , IL-6 ↓	miR-150-5p/MEKK3/JNK/jun	Inhibited RGC apoptosis, M1 to M2 shift	Wang et al. (2023)
hBMSC-EV	IRI	RGCs	caspase-3 ↓ , capsase-7 ↓ , Bax ↓ , Bcl-2 ↓ , p53 ↓ , TNF- α↓ , VEGF ↓ , IL-1 β↓ , iNOS ↓ , ICAM-1 ↓ , MCP1 ↓	miR-424	Protected against hypoxia and inflammation	Mathew et al. (2023)
hBMSC-EV	IRI	Retinal neurons and RGCs	TNF- α↓ , IL-6 ↓ , caspase-3 ↓	Not reported	Inhibited apoptosis, downregulated cytokines	Mathew et al. (2019)
rat BMSC-exo	RDD	Photoreceptors, microglia	IL-1 β↓ , TNF- α↓ , IL-6 ↓ , iNOS ↓	Not reported	Protected photoreceptors, inhibited microglia	Luodan et al. (2024)
mBMSC-exo	RDD	Photoreceptors	PDCD-4 ↓	miR-21/PDCD-4	Inhibited apoptosis, protected retina	Deng et al. (2021)
hBMSC-exo	RDD	Photoreceptors	Not reported	Not reported	Protected photoreceptors	Park et al. (2021)
mBMSC-exo	DR	NFL, GCL, INL and OPL	Phosphorylated β -catenin ↑ , Nox2 ↓ , Nox-4 ↓ , SOD1 ↑ , SOD2 ↑ , c-Myc ↓ , cyclinD1 ↓ , TNF- α↓ , ICAM-1 ↓ , VEGF ↓	Wnt/ β -catenin	Ameliorated oxidative stress, suppressed inflammation, attenuated angiogenesis and vascular leakage	Ebrahim et al. (2022)
mBMSC-exo	DR	HRMECs	SOD ↑ , CAT ↑ , GSH-Px ↑ , MDA ↓ , VEGF ↓ , Cox-2 ↓ , MMP-2 ↓ , ICAM1 ↓	miR-133b-3p/FBN1	Regulated angiogenesis, suppressed ROS	Liang et al. (2022)
hBMSC-exo	DR	HRMECs	XBP1 ↑ , miR-34a-5p ↓	SNHG7/miR-34a-5p/XBP1	Suppressed EMT and tube formation	Cao et al. (2021)
mBMSC-exo	DR	M Uller cells	miR-486-3p ↓ , TLR4 ↓ , NF- κ B ↓ , VEGF ↓ , MMP-9 ↓ , VCAM-1 ↓ , COX-2 ↓	miR-486-3p/TLR4/NF- κ B	Suppressed inflammation, promoted proliferation	Li et al. (2021)
hBMSC-exo	OIR	ILM	Not reported	Not reported	Reduced retinal thinning, inhibited neovascularization	Moisseiev et al. (2017)
hBMSC-EV	Corneal wound	HCEs	BAD ↓ , P53 ↓ , BCL-2 ↓ ; caspase-3 ↓	Not reported	Inhibited apoptosis	Tati et al. (2024)
hBMSC-EV	Corneal wound	HCEs	TNF- α↓ , IL-1 β↓ , IL-6 ↓ , Spp1 ↑ ; Flt-1 ↓ , Kdr ↓ , Flt-4 ↓ , VEGF- α↓ , VEGF-d ↓	TGF- β	Downregulated inflammation, suppressed angiogenesis	Saccu et al. (2022)
mBMSC-exo	RD	RGCs	IL-1 β↓ , TNF- α↓ , LC3-II ↑ , Atg-5 ↑	Not reported	Inhibited apoptosis, improved autophagy	Ma et al. (2020a)

Abbreviations: BMSC-exos, Bone Marrow Stromal Cell-derived exosomes; RGCs, Retinal Ganglion Cells; ONC, Optic Nerve Crush; GLC, Glaucoma; IRI, Ischemia-Reperfusion Injury; RDD, Retinal Degenerative Disease; DR, Diabetic Retinopathy; OIR, Oxygen-Induced Retinopathy; RD, Retinal Detachment; iv, intravenous; ivt, intravitreal; sub, subretinal; top, topical; NR, Not Reported; Dis, Disease; Ref, Reference; casp, caspase; p- β -cat (phosphorylated β -catenin), ↑ (upregulated), ↓ (downregulated).

### 5.1 Optic nerve injury

In traumatic optic neuropathy, major causes of blindness include RGC loss and axonal degeneration. Current treatments are limited, however, and often rely on injections of neuroprotective agents, including NGF and BDNF (Mohammad et al., 2018). These agents mitigate damage by promoting nerve cell growth, repair, RGC survival, and axon regeneration (Andrew et al., 2018).

Research by Mead and Tomarev. (2017) demonstrated that BMSC-exos promote neuroprotection and neuritogenesis in primary RGC cultures in ONC model rats. Quantitatively, treatment with BMSC-exos significantly preserved retinal nerve fibre layer (RNFL) thickness, which decreased only to 33.8±4.8 μm (from a baseline of 48.4±2.9 μm ) compared to a more severe reduction to 18.0±2.1 μm in the control group (from a baseline of 48.2±1.3 μm , p<0.05 ). These structural improvements were associated with the preservation of the functionality of over 50 % of the RGCs and improvements of axonal growth length to 114.2±5 μm , which was approximately 2.5 times greater than that in the control group ( 43.7±6.9 μm , p<0.05 ).

Previous studies have proposed that this effect is due to the rich secretion of NGFs, such as platelet-derived growth factor and BDNF (Mead et al., 2013), by BMSC-exos Mead et al. (2014) Furthermore, Cui et al. (2021) demonstrated that BMSC-exos exert antiapoptotic effects in a rat ONC model by suppressing the expression of proapoptotic proteins (Bax and caspase-3) and increasing the levels of antiapoptotic Bcl-2 via the PI3K/Akt signalling pathway. Additionally, BMSC-exos were shown to downregulate inflammatory cytokines such as TNF- α , IL-1 β , IL-6, IL-8 and MCP-1. Using systematic KEGG analysis, You et al. (2025) investigated the molecular mechanisms underlying the neuroprotective and reparative effects of BMSC-exos in ONC rat models and revealed that they (less than 220 nm) significantly regulate key proinflammatory pathways (IL-17, TNF, NF- κ B, and HIF-1 pathways) and that ribosome-related pathways are significantly differentially modulated.

Furthermore, He et al. (2018) confirmed that BMSC-exos downregulate VEGF-A expression in ARPE-19 cells within laser-injured retinas; compared with those in model groups, increasing the BMSC-exo concentration progressively decreased the VEGF-A mRNA and protein levels, suggesting an anti-inflammatory role through a reduction in the levels of inflammatory cytokines.

In glaucoma models, Mead et al. (2018a) demonstrated that BMSC-exos deliver NGF and BDNF to RGCs, reducing the number of severe degenerating axons (by 40 % ) compared with control group (by 66 % , p<0.05 ). Additionally, Mead and Tomarev (2017) suggested that BMSC-exos contain miRNAs that may promote RGC regeneration. RNA sequencing revealed that, compared with fibroblast-exos, BMSC-exos were enriched in miR-17-92, miR-21 and miR-146a. miR-17-92 and miR-21 downregulate PTEN, whereas miR-146a affects the concentrations of epidermal growth factor receptor (EGFR), both of which are normally involved in suppressing RGC axon growth and survival. These authors further demonstrated that BMSC-exos inhibited RGC apoptosis and loss, preserved RGC function during early decline phases, and prevented RNFL degeneration (e.g., pSTR amplitudes in BMSC-exo treated 24.9±2.4 μV compared with control gooup 18.5±2.2 μV , p<0.05 ) (Mead et al. 2018a; Mead et al., 2018b).

In summary, BMSC-exos containing miRNAs and signalling molecules provide sustained synergistic neuroprotection in optic nerve injury and glaucoma models by counteracting inhibitory factors, reducing inflammation and suppressing apoptosis. These findings support the potential effectiveness, safety and long-acting neuroprotective and neuroregenerative effects of BMSC-exos in treating RGC injury and disorders, though translating these promising neuroprotective effects to human optic neuropathies requires overcoming challenges in achieving sustained RGC targeting and demonstrating efficacy in complex human disease contexts beyond acute injury models.

### 5.2 Retinal degenerative diseases

RDDs, which include conditions such as retinitis pigmentosa (RP) and AMD, are characterized by the progressive loss of retinal neurons, particularly photoreceptors, leading to irreversible vision impairment. Current therapeutic strategies, including gene therapy, anti-VEGF agents, and neuroprotective compounds, have disadvantages such as limited applicability, the need for repeat administration, or relatively poor efficacy (Reza et al., 2025). Therefore, there is a great need to investigate novel treatments such as BMSC-exos.

Accumulating evidence has demonstrated the therapeutic potential of BMSC-exos in various RDD models. Deng et al. (2021) reported that BMSC-exos containing miR-21 suppressed PDCD4 expression, thereby protecting photoreceptors from apoptosis and mitigating retinal degeneration in both MNU-induced RDD and RP models (e.g., reduce the decrease in b-wave amplitude over 50 % compared with control group, p<0.01 ). Park et al. (2021) demonstrated that BMSC-exos exert neuroprotective effects in a Royal College of Surgeons (RCS) rat model of retinal degeneration. Furthermore, An et al. (2025) reported that BMSC-exos promote photoreceptor survival by inhibiting microglial overactivation and modulating the retinal immune microenvironment, specifically, through suppression of the expression of IL-1 β , TNF- α , IL-6, and iNOS. Similarly, recent research has demonstrated that BMSC-exos delayed photoreceptor degeneration in an RP mouse model by downregulating inflammatory cytokines (IL-6, TNF- α , and IL-1 β ) and suppressing neuroinflammation (Massoumi et al., 2023). However, translating these promising results into human clinical applications, demonstrating that BMSC-exos therapy can significantly halt degeneration in chronic human disease settings, and defining the optimal treatment window, are critical next steps.

### 5.3 Ischemic retinopathy

IRI is a common pathophysiological process in ischaemic retinal diseases, caused by temporary blockage and reperfusion of blood flow. The mechanism by which IRI causes tissue injury is complex and includes endothelial damage, inflammatory responses, and apoptosis.

Several studies have shown that BMSC-exos downregulate inflammatory cytokines and suppress apoptosis to protect the retina from ischaemic damage. Mathew et al. (2019) demonstrated that BMSC-exos reduce the occurrence of cell death in R28 cells subjected to oxygen and glucose deprivation (OGD) *in vitro*, attenuate retinal neuronal apoptosis (reduction of more than 50 % in TUNEL-positive cells compared to the control group, p<0.05 ) and the inflammatory mediators IL-6 and TNF- α , and reduce cell death in RGCs and microglia *in vivo* in a model of retinal ischaemia. Additionally, these authors demonstrated that hypoxic preconditional BMSC-exos and miR-424-overexpressing BMSC-exos downregulated proapoptotic genes such as those encoding caspase-3, capsase-7, Bax and p53; inhibited RGC apoptosis; and prevented RGCs from undergoing hypoxia and inflammation in a high-intraocular pressure (IOP)-induced IRI rat model (Mathew et al., 2023). Wang et al. (2023) designed Sca-1+ BMSC-exos to reduce M1 microglial polarization by over 50 % (p<0.01) , promote RGC survival and prevent the reduction of thickness (approximately 50 % reduction, p<0.01 ) in multiple layers of the retina via the miR-150-5p/MEKK3/JNK/c-jun pathway in an IRI rat model. A study on gingival MSCs separately revealed the protective effects of these MSCs against IRI-induced retinal damage and demonstrated that miR-21-5p contained in gingival MSC exosomes regulates the expression of PDCD4 to exert antiapoptotic effects on RGCs and inhibit inflammation via neuroprotective mechanisms (Yu et al., 2016). Notably, miR-21-5p is also enriched in BMSC-exos Baglio et al. (2015), suggesting their potential role in neuroprotection against IRI. Thus, developing BMSC-exos to deliver this and other neuroprotective miRNAs may mitigate retinal neuronal damage following ischaemic events.

These studies indicate that BMSC-exos have significant therapeutic potential for treating retinal ischaemic diseases. Their miRNA and protein cargoes can modulate inflammatory pathways and inhibit apoptotic cascades, thereby alleviating retinal ischaemic injury though clinical translation requires further validation of efficacy and safety.

### 5.4 Diabetic retinopathy

DR, one of the most serious microvascular complications of diabetes, is a leading cause of blindness. Existing studies have suggested that DR results from glucose metabolism disorders, haemodynamic changes, local hypoxia and inflammation secondary to vascular dysfunction. Standard treatments for DR include laser photocoagulation, vitrectomy, and the intravitreal administration of anti-VEGF agents (Reddy et al., 2023).

Several recent *in vitro* experimental studies have demonstrated that BMSC-exos cocultured with high-glucose (HG)-treated retinal cells can exert therapeutic effects through multiple miRNA pathways. Cao et al. (2021) demonstrated that BMSC-exos containing SNHG7, a long noncoding RNA reported to repress HG-induced angiogenesis in human retinal microvascular endothelial cells (HRMECs) via the miR-543/SIRT1 pathway, are overexpressed in HRMECs and can suppress HG exposure-induced endothelial-to-mesenchymal transition and angiogenesis (reduction of more than 35 % tube formation, p<0.01 ) through the miR-34a-5p/XBP1 axis. Similarly, Li et al. (2021) confirmed that BMSC-exos containing miR-486-3p inhibit oxidative stress, inflammation and apoptosis while promoting M uller cells proliferation by more than 30 % relative to the untreated controls (p<0.01) and suppressing angiogenesis by downregulated cytokines (e.g., VEGF, MMP-9, VCAM-1, COX-2) in HG-treated M uller cells via the miR-486-3p/TLR4/NF- κ B signalling pathway. Additionally, Liang et al. (2022) demonstrated that BMSC-Exos effectively suppress expression of major molecules associated with pathological angiogenesis (e.g., VEGF, COX-2, MMP-2, ICAM-1) in HG-treated mouse retinal microvascular endothelial cells (mRMECs). That study revealed that BMSC-exos significantly inhibit cellular proliferation and migration while promoting apoptosis through the upregulation of miR-133b-3p and the concurrent downregulation of FBN1, which attenuate the elevated expression of inflammatory mediators, including VEGF and COX-2. Similarly, Ebrahim et al. (2022) demonstrated that BMSC-exos ameliorate oxidative stress by downregulating Nox2/Nox4 mRNA (by 50 % compared with control, p<0.05 ) and NOX1 protein (by 75 % compared with control, p<0.05 ), while upregulating SOD1/SOD2 mRNA (by 30 % compared with control, p<0.05 ), suppress inflammation by reducing TNF- α and ICAM-1 (by 50 % compared with control, p<0.05 ), and attenuate pathological angiogenesis by decreasing VEGF mRNA (by 25 % compared with control, p<0.05 ) and protein (by 50 % compared with control, p<0.05 ) in a streptozotocin (STZ)-induced hyperglycaemic rat model. These effects, confirmed via RT-PCR, ELISA, enzymatic assays, and immunohistochemistry, were mediated through the inhibition of the Wnt β -catenin signaling pathway.

These protective effects occur through suppression of the Wnt/ β -catenin signalling pathway, leading to downregulation of oxidases, adhesion molecules and inflammatory factors (Ebrahim et al., 2022).

### 5.5 Optic neuritis

Optic neuritis (ON) is associated with several systemic autoimmune disorders. The pathological changes in ON include inflammation, demyelination of the optic nerve and damage to the inner retina, which can result in reduced visual quality, vision loss, and even blindness. Current available treatments include steroid administration, chemotherapy and autologous haematopoietic stem cell transplant or immune reconstitution therapy (Aneesh et al., 2021).

Owing to their immunomodulatory and neuroregenerative properties, BMSC-exos may have therapeutic potential for ON. A recent study demonstrated that BMSC-exos protect cells from apoptosis in experimental ON models, potentially via the delivery of neurotrophic factors such as GDNF and CNTF (Massoumi et al., 2023).

A recent study revealed that BMSC-exos suppressed T-cell proliferation and regulatory T cells (Tregs) induction by regulating microglial activity and promoting a shift in the microglial phenotype from the M1 (proinflammatory) phenotype to the M2 (anti-inflammatory) phenotype, decreasing the degrees of neuroinflammation and demyelination (Riazifar et al., 2019). These findings suggest that exos could provide a cell-free alternative to stem cell transplantation for promoting remyelination and axonal preservation in patients with inflammatory optic neuropathies.

### 5.6 Retinopathy of prematurity

Retinopathy of prematurity (ROP) is a proliferative retinopathy that affects vision in premature infants and can potentially cause blindness. Current treatments for ROP include laser treatment, vitrectomy, and intravitreal injections of anti-VEGF agents (Dammann et al., 2023). A recent study demonstrated that the intravitreal injection of human BMSC-exos preserves retinal vascular flow, reduces neovascularization, and mitigates retinal thinning in an oxygen-induced retinopathy (OIR) mouse model simulating ROP. Proteomic analysis of BMSC-exos revealed the presence of prosurvival proteins, including components of the cAMP response element-binding protein (CREB) signalling pathway (Moisseiev et al., 2017).

### 5.7 Retinal detachment

Retinal detachment (RD) is characterized by separation of the retinal neurosensory layer from the RPE. This condition causes retinal ischaemia and hypoxia, which in turn induce photoreceptor apoptosis and vision decline. Ma et al. (2020a) investigated the therapeutic effects of BMSC-exos in an RD rat model and found that their administration significantly reduces the expression of inflammatory factors (TNF- α and IL-1 β ), decreases photoreceptor apoptosis (approximately 80 % decrease in TUNEL-positive cells compared with PBS-treated group, p<0.05 ) via enhanced the autophagy pathway and preserves retinal structural integrity.

## 6 Conclusion

In conclusion, BMSC-exos represent a promising preclinical strategy by enabling multifaceted interventions for debilitating ocular diseases. These nanovesicles may bridge critical gaps in the treatment of neuro-ophthalmic disorders where current strategies–including neuroprotective agents, anti-VEGF therapies, and surgery–demonstrate limited efficacy, narrow applicability, and poor patient compliance due to frequent dosing or inadequate neurorestorative effects. BMSC-exos deliver a heterogeneous bioactive cargo that improves neuroprotection (e.g., promoting RGC survival in glaucoma models; preserving photoreceptors in retinal detachment), suppresses inflammatory responses (e.g., reducing TNF- α and IL-1 β in ischaemic retinopathy), and stimulates regenerative processes (e.g., via miR-17-92-mediated axon regrowth). Although these findings highlight the therapeutic potential of BMSC-exosomes, their translational viability remains to be proven in human subjects.

Despite encouraging outcomes in rodent models, the translation of BMSC-exosome therapy into clinical practice has been significantly impeded. This “translational gap” is driven by intrinsic biological complexities and insufficient safety data, necessitating critical attention to the following challenges:

1. The Preclinical Model Dilemma: A fundamental limitation is the predominant reliance on acute rodent models, which fail to recapitulate the chronic, complex pathophysiology of human retinal diseases such as AMD and glaucoma. While mechanistically informative, data derived from these small-animal models often offer an overly optimistic forecast of clinical success. Crucially, the paucity of data from large animal models (e.g., non-human primates)–which share anatomical and physiological similarities with the human eye–prevents a realistic assessment of long-term efficacy and pharmacokinetics. Without validation in substantial model systems, the therapeutic benefits remain theoretical.
2. Unknown Risks and Safety Profiles: The safety profile of BMSC-exos is far from established. The intraocular injection of heterogeneous exosome populations carries risks of inflammation, fibrosis, or autoimmune response due to the presence of uncharacterized cytokines or miRNAs Ye et al. (2025a), Bai et al. (2017). (1) Regulatory Hurdles: Current safety data are largely derived from short-term (e.g., 28-day) observations. Regulatory approval requires rigorous Good Laboratory Practice (GLP)-compliant toxicology studies, including maximum tolerated dose (MTD) determination and long-term biodistribution profiling, which are currently lacking. Table 3 lists the doses of exosomes used and the number of observation days in different disease models in recent studies. However, toxicological experiments such as the MTD still need to be further conducted. (2) Absence of Clinical Trials: Crucially, there are no active interventional clinical trials investigating MSC-exos for retinal or optic nerve diseases. A review of the ClinicalTrials.gov database identifies only five relevant entries, none of which provide robust evidence for retinal application. Two studies–one targeting macular holes (NCT03437759) and another regarding retinitis pigmentosa (NCT05413148)–are currently listed with an “unknown” status. The remaining three studies focus exclusively on low-risk ocular surface diseases using topical administration. The absence of patient recruitment for retinal indications underscores the consensus that insufficient model system data exists to justify the efficacy of intravitreal administration in humans.
3. Inherent Heterogeneity and Manufacturing Instability: A pervasive barrier to clinical utilization is the tremendous heterogeneity of exosome preparations Li et al., 2023. This not only confounds experimental reproducibility but also poses a major barrier to the scalable, consistent, and cost-effective production of clinical-grade exosomes. (1 Source Variability: Exosomes secreted by MSCs of distinct origins (e.g., ADSCs, UCMSCs, and GMSCs) exhibit differences in cargo and functional characteristics, reflecting the specific features of their parental cells. A recent study also suggests sexual heterogeneity in therapeutic potency, where exosomes from female donors demonstrated superior efficacy in inhibiting RGC apoptosis compared to male donors in ischaemic models (Yuan and Yu, 2016). (2) Microenvironmental Influence: The characteristics and functions of exosomes are dynamically influenced by the bidirectional communication between their parental cells and the local microenvironment. Disease-specific conditions (e.g., inflammation, ischemia, fibrosis) may alter the protein and miRNA composition of MSC-Exos, thereby unpredictably influencing their therapeutic effectsYe et al. (2025b). (3) Compositional Inconsistency: MSC-Exos are nanoscale vesicles rich in bioactive molecules, including proteins, lipids, and nucleic acids. The specific composition and proportion of these molecules may vary significantly among different batches or under different culture conditions. This variability directly leads to heterogeneity in biological effects (e.g., immune regulation, angiogenesis promotion, and tissue repair), complicating standardization.
4. Unsolved Delivery Challenges for Retinal Targets: Achieving efficient, sustained, and cell-specific delivery to the retina and optic nerve while minimizing off-target exposure remains a significant engineering hurdle. Current injection-based methods are suboptimal, and innovative formulations (e.g., hypoxia preconditioning, genetic engineering) or novel delivery systems are required to maximize the therapeutic index.

**TABLE 3:** Therapeutic applications and mechanisms of BMSC-derived exosomes in retinal and optic nerve pathologies.

Type of EV	Model	Administration	Frequancy	Reference
Rat BMSC-exo	rat ONC model via crushing	$1\times 10^{6}$ in 1 µL intravitreally injected	Once within 21 days	You et al. (2025)
mBMSC-exo	rat ONC model via crushing	$3\times 10^{9}$ in 5 µL iv	Once within 30 days	Cui et al. (2021)
hBMSC-exo	rat ONC model via crushing	$3\times 10^{9}$ in 5 µL iv	Once-per-week for 3 weeks	Mead and Tomarev. (2017)
hBMSC-sEV	Genetic DBA/2J mice	$1\times 10^{9}$ in 2 µL iv	Once within 21 days	Mead et al. (2018a)
hBMSC-sEV	Ocular hypertension rats	$3\times 10^{9}$ in 5 µL iv	Once-per-month for 9 months	Mead et al. (2018b)
Sca-1+ mBMSC-exo	High IOP-induced IRI mice	2 µL iv	Once within 7 days	Wang et al. (2023)
hBMSC-EV	High IOP-induced IRI rats	$4\times 10^{6}$ in 4 µL iv	Once within 8 days	Mathew et al. (2023)
rat BMSC-exo	RCS rat models	$1\times 10^{11}$ iv	Once within 21 days	Luodan et al. (2024)
mBMSC-exo	MNU-induced rat model	1 µg in 1 µL iv	Once within 8 weeks	Deng et al. (2021)
hBMSC-exo	RCS rat model	10 µg in 4 µL subretina/iv	Once within 7 weeks	Park et al. (2021)
mBMSC-exo	DM rat model of STZ-induced hyperglycaemia	50 µg in 500 µL intravitreally injected	Once within 4 weeks	Ebrahim et al. (2022)
hBMSC-exo	Hyperoxic conditioning induced IRI mice	20 µg in 1 µL iv	Once within 2 weeks	Moisseiev et al. (2017)
mBMSC-exo	Hyaluronic acid induced RD rats	100 µg in 5 µL subretina	Once within 7 days	Ma et al. (2020a)

Abbreviations: BMSC-exo, Bone Marrow Stromal Cell-derived exosomes; ONC, Optic Nerve Crush; IRI, Ischemia-Reperfusion Injury; RCS, Royal College of Surgeons; MNU, N-Methyl-N-Nitrosourea; STZ, Streptozotocin; OIR, Oxygen-Induced Retinopathy; RD, Retinal Detachment; iv, intravenous; ivt, intravitreal.

Future Directions Addressing these limitations through targeted research is crucial for translating the potential of BMSC-exos into safe and effective clinical treatments. Future research should focus on the following directions:

Developing Predictive Preclinical Platforms: Prioritize and invest in chronic disease models in non-human primates or other large animals that better simulate human disease pathophysiology. Conducting Regulatory-Driven Safety Studies: Perform rigorous GLP toxicology and biodistribution studies specifically addressing potential risks (e.g., immunogenicity, tumorigenicity) identified in other fields, thereby establishing a solid safety foundation for IND applications. Advancing from Descriptive to Functional Mechanistic Studies: Employ targeted approaches (e.g., hypoxic environment, cytokine-stimulated MSCs) to identify critical active components. This knowledge will drive the development of potency assays and engineered exosomes with enhanced and reproducible functionality. Standardizing and Characterizing Heterogeneity: Move beyond basic characterization to functional potency assays. Investigate the impact of donor variables and develop engineering strategies to produce homogeneous, reproducible exosome populations. Exploring Rational Combination Therapies: Investigate synergies with existing standards of carrier (e.g., anti-VEGF agents). Exosome-mediated drug delivery systems represent a pragmatic approach to potentially improve efficacy and accelerate clinical adoption. Innovating in Formulation and Delivery: Develop novel delivery platforms to enhance targeting and prolong retinal residence time, thereby improving the therapeutic window.
